# Supplementary material for: Relationship between Seed Morphological Traits and Ash and Mineral Distribution along the Kernel Using Debranning in Durum Wheats from Different Geographic Sites
Source: Foods. 2020 Oct 23;9(11):1523. doi: 10.3390/foods9111523 (PMC7690802; doi:10.3390/foods9111523)
Supplement: Supplementary file 1 [file foods-09-01523-s001.pdf]

Table S1 (Supplemental Material). Milling (wholemeal and semolina) and debranned kernel flours (DK1-DK6) of four genotypes over three environments: quantification of ash and macro-elements (a) and micro-elements (b).

a)

Genotype	Environment	Treatment	Ash	Na	K	P	Ca	Mg	Sum macro-
	Palermo	WM	2.1	411.86	5339.82	3900.62	705.76	1359.99	14366.7
		DK1	1.9	311.65	5077.14	3609.77	634.77	1328.77	13525.6
		DK2	1.8	290.34	4672.18	3425.53	607.90	1203.10	12655.0
		DK3	1.6	277.44	4069.31	2901.88	539.99	1017.06	11250.4
		DK4	1.5	273.29	3719.02	2496.76	465.21	884.23	10085.7
		DK5	1.5	264.28	3783.56	2257.94	356.38	897.18	9677.4
		DK6	1.3	262.76	3658.51	1528.85	231.52	854.17	8430.2
		S	0.8	221.63	2218.37	1107.56	204.79	466.02	6098.6
Russello	Agrigento	WM	2.1	394.39	5213.51	3237.69	830.23	1246.24	13383.4
		DK1	1.9	309.72	4620.15	3223.59	803.61	1160.07	11948.7
		DK2	1.7	304.99	4103.63	2561.79	794.32	979.36	11237.3
		DK3	1.6	284.95	3977.39	2188.81	695.96	929.12	14365.9
		DK4	1.5	283.70	4075.06	1742.51	639.32	965.96	10696.4
		DK5	1.5	283.23	3649.50	1486.81	640.95	811.32	9740.0
		DK6	1.3	279.17	3464.61	1135.08	530.69	764.66	8972.5
		S	0.9	255.70	2147.84	657.00	497.20	416.95	6726.7
	Foggia	WM	2.0	297.05	4631.39	2792.94	816.98	1247.69	13489.8
		DK1	1.8	278.51	3975.27	2462.62	741.65	1059.51	12178.7
		DK2	1.6	278.04	3736.21	2173.66	748.37	973.70	11543.9
		DK3	1.5	277.07	3501.61	2022.03	713.08	921.32	11024.1
		DK4	1.4	245.24	3214.52	1576.72	705.55	792.49	10069.3
		DK5	1.3	243.34	2846.49	1502.33	617.23	725.64	9458.6
		DK6	1.2	241.53	2899.22	1272.48	575.83	670.09	9090.5
		S	0.9	236.52	2168.90	603.41	568.72	437.07	7273.3
	Palermo	WM	2.0	279.22	5290.63	3936.82	652.32	1438.88	14837.3
		DK1	1.7	278.14	4250.52	2800.01	595.47	1160.98	12292.8
		DK2	1.5	272.18	3816.69	2143.63	597.69	1029.11	10854.4
		DK3	1.4	268.07	3458.19	2007.06	489.99	860.24	10021.6
		DK4	1.2	257.72	3312.82	1848.79	441.07	810.31	9541.1
		DK5	1.2	256.59	3097.39	1761.25	438.09	720.01	8989.7
		DK6	1.1	255.31	2888.08	1526.39	425.17	663.10	8456.5

		S	0.9	254.04	2565.31	1432.57	300.59	600.89	7832.6
Perciasacchi	Agrigento	WM	2.1	277.25	5053.96	3260.55	582.57	1113.05	12891.6
		DK1	1.7	273.69	5034.31	2476.21	547.28	1061.31	11972.1
		DK2	1.5	260.84	4018.11	2156.35	410.95	907.92	10327.1
		DK3	1.3	257.62	3627.42	2175.67	335.45	751.37	9711.6
		DK4	1.2	256.66	3511.09	1998.73	330.89	686.60	9320.9
		DK5	1.1	255.46	3282.02	1929.59	347.97	623.48	8953.5
		DK6	1.1	255.06	3082.30	1615.00	340.68	591.90	8390.2
		S	1.0	249.17	2604.91	1183.02	275.66	488.58	7180.4
	Foggia	WM	2.0	294.82	4731.25	2761.97	593.38	996.80	12518.6
		DK1	1.7	289.31	4254.52	2590.99	541.85	925.26	11702.3
		DK2	1.5	281.49	3978.00	2313.54	481.08	823.02	10947.7
		DK3	1.3	279.36	3505.71	1821.00	461.66	683.77	9763.7
		DK4	1.2	271.32	3360.23	1594.60	361.17	628.36	9200.2
		DK5	1.1	270.45	2942.28	1238.40	345.28	505.87	8100.0
		DK6	1.1	267.06	3007.72	1170.44	341.06	489.35	8071.6
		S	1.0	265.27	2619.89	944.78	318.06	522.90	7338.4
	Palermo	WM	2.2	351.39	5327.76	3633.41	657.15	1495.82	13762.7
		DK1	2.0	314.24	4504.06	3330.84	612.58	1329.61	12301.4
		DK2	1.9	301.44	4430.81	3228.69	580.85	1335.47	12140.2
		DK3	1.8	290.78	3927.40	2701.80	592.18	1152.43	10896.8
		DK4	1.6	281.16	3750.34	2723.13	511.89	1120.75	10531.2
		DK5	1.5	280.60	3524.23	2368.48	503.48	1011.25	9827.0
		DK6	1.5	273.48	3599.14	2282.83	405.23	995.49	9626.6
		S	0.7	273.17	1521.68	348.30	375.11	391.79	4849.7
Timilia	Agrigento	WM	2.0	350.74	4889.44	3301.18	538.19	1381.59	13848.9
		DK1	1.9	307.48	4399.82	3137.73	460.99	1311.51	12929.3
		DK2	1.7	301.56	3915.76	2748.54	459.55	1162.61	11795.9
		DK3	1.6	290.44	3526.84	2010.76	446.75	990.43	10329.5
		DK4	1.5	283.77	3542.67	2072.15	405.37	981.72	10267.7
		DK5	1.4	281.20	3152.21	1637.24	320.93	815.87	9034.4
		DK6	1.3	270.18	2877.92	1350.15	267.50	750.87	8238.9
		S	0.6	260.68	1447.36	109.41d	283.09	321.88	5085.5
	Foggia	WM	1.9	339.05	4421.22	4386.47	325.71	1316.78	13026.8
		DK1	1.8	294.31	4304.88	4087.85	350.12	1283.31	12305.4
		DK2	1.7	290.43	3944.68	3847.06	304.89	1178.13	11531.1
		DK3	1.6	283.49	3696.22	3811.37	319.88	1102.61	11030.2
		DK4	1.5	271.79	3246.04	3088.40	333.77	899.40	9629.8

		DK5	1.4	265.09	3017.98	2984.15	285.38	861.56	8970.2
		DK6	1.3	264.85	2921.17	2526.55	188.75	780.81	8228.1
		S	0.7	261.41	1467.37	1449.31	174.83	327.81	5110.2
	Palermo	WM	2.3	432.07	5265.93	3870.67	596.59	1408.58	15161.4
		DK1	1.9	404.57	4673.99	3640.20	425.41	1230.35	13885.3
		DK2	1.8	372.49	4089.46	3245.53	344.35	1122.59	12455.2
		DK3	1.6	358.57	3643.15	2973.03	304.61	969.54	11473.7
		DK4	1.5	355.60	3503.38	2495.48	283.97	957.84	10697.2
		DK5	1.4	348.07	3242.21	2275.59	233.05	837.70	9719.4
		DK6	1.2	342.19	2852.72	1977.26	222.41	703.33	8762.1
		S	1.0	327.59	2126.6	1409.85	200.27	552.10	7211.9
Cappelli	Agrigento	WM	2.0	395.94	4981.69	3374.40	485.56	1273.12	14876.2
		DK1	1.8	392.05	4395.09	3264.36	325.00	1173.76	13492.2
		DK2	1.6	387.31	3594.53	2390.87	255.59	936.47	11394.6
		DK3	1.4	363.76	3422.17	2261.94	263.48	859.57	10979.1
		DK4	1.3	362.83	2939.56	1733.82	254.87	716.46	9723.3
		DK5	1.2	362.07	2780.25	1642.40	221.43	657.01	9338.5
		DK6	1.1	358.65	2782.38	1661.38	201.58	662.47	9269.9
		S	0.8	341.53	1843.21	810.56	164.96	396.42	7092.4
	Foggia	WM	2.1	457.55	4667.34	3252.42	453.78	1242.53	13905.7
		DK1	1.8	400.18	4368.12	3005.36	451.49	1129.86	12918.4
		DK2	1.5	397.03	3434.82	2244.85	368.14	895.50	10794.1
		DK3	1.3	376.95	3091.21	1804.79	334.25	773.08	9726.4
		DK4	1.2	376.71	2818.14	1635.46	308.45	704.28	9181.9
		DK5	1.1	365.37	2704.08	1480.09	255.77	651.11	8745.5
		DK6	1.1	361.98	2581.32	1285.91	196.56	613.46	8313.1
		S	0.8	340.80	1992.2	781.11	130.78	476.85	6985.9

Analysis of variance	*F*	*p*	*F*	*p*	*F*	*p*	*F*	*p*	*F*	*p*	*F*	*p*	*F*	*p*
G	256.6	***	723.8	***	53.3	***	86.7	***	623.7	***	143.5	***	51.9	***
E	251.5	***	1.7	ns	124.8	***	96.8	***	8.8	***	170.9	***	51.8	***
T	3023	***	106.5	***	897.1	***	452.5	***	214.8	***	637.2	***	1086.8	***
G x E	46.1	***	19.5	***	21.8	***	80.6	***	138.4	***	2.9	*	5.8	***
G x T	44.1	***	6.5	***	11.1	***	8.4	***	5.6	***	11.7	***	13.0	***
E x T	3.2	***	1.5	ns	4.4	***	1.0	ns	3.7	***	2.5	**	3.5	***
G x E x T	2.1	**	1.9	**	2.2	***	2.3	***	2.3	***	1.8	*	2.5	***

b)

Genotype	Environment	Treatment	Mn	Fe	Cu	Zn	Mo	Sum micro-
Russello	Palermo	WM	38.73	55.06	7.29	34.41	1.59	137.1
		DK1	32.31	49.74	6.87	33.08	1.71	123.7
		DK2	28.26	48.18	6.42	29.23	1.59	113.7
		DK3	24.85	43.45	6.31	28.40	1.49	104.5
		DK4	24.86	43.15	5.90	27.41	1.49	102.8
		DK5	21.47	39.43	5.73	26.33	1.47	94.4
		DK6	21.13	36.32	4.81	22.97	1.40	86.6
		S	8.12	28.54	4.29	15.61	1.38	57.9
	Agrigento	WM	31.16	50.36	5.99	42.64	5.31	135.5
		DK1	23.72	41.38	5.64	34.00	4.92	109.7
		DK2	20.28	37.24	5.21	31.73	4.84	99.3
		DK3	20.06	32.33	5.12	30.52	4.68	92.7
		DK4	19.67	30.03	5.00	27.57	4.54	86.8
		DK5	17.61	30.04	4.22	25.68	4.45	82.0
		DK6	16.21	24.23	4.05	22.04	4.34	70.9
		S	6.77	22.31	3.33	15.38	3.77	51.6
	Foggia	WM	48.29	35.98	4.96	39.90	2.35	131.5
		DK1	36.47	31.87	5.24	33.04	2.20	108.8
		DK2	32.02	28.64	5.00	31.48	2.18	99.3
		DK3	31.41	28.38	4.52	28.06	2.13	94.5
		DK4	28.54	27.69	3.88	21.54	2.03	83.7
		DK5	26.91	24.87	3.73	21.48	1.97	79.0
		DK6	25.92	23.48	3.48	18.94	1.91	73.7
		S	11.66	22.98	3.16	11.74	1.83	54.1
Perciasacchi	Palermo	WM	36.04	57.88	6.95	43.08	0.68	144.6
		DK1	29.67	49.77	6.05	33.02	0.58	119.1
		DK2	22.68	48.00	5.87	32.10	0.55	109.2
		DK3	21.42	40.37	5.56	28.23	0.55	96.1
		DK4	19.35	38.76	5.18	24.34	0.50	88.1
		DK5	19.42	35.75	4.69	23.80	0.45	84.1
		DK6	19.94	32.04	4.40	18.24	0.42	75.0
		S	12.22	31.36	4.18	15.14	0.39	63.3
	Agrigento	WM	24.74	39.77	5.61	54.44	4.23	128.8

		DK1	20.83	33.40	5.23	42.58	4.05	106.1
		DK2	16.85	29.25	5.23	39.57	3.94	94.8
		DK3	14.54	30.37	5.01	38.71	3.84	92.5
		DK4	16.33	28.45	5.06	38.53	3.85	92.2
		DK5	16.18	28.08	4.55	36.80	3.77	89.4
		DK6	14.24	23.63	4.47	28.15	3.74	74.2
		S	7.77	20.62	3.53	21.32	3.64	56.9
	Foggia	WM	38.61	43.92	5.48	57.54	1.76	147.3
		DK1	31.56	32.5	4.82	44.31	1.49	114.8
		DK2	31.30	30.81	4.80	40.64	1.40	109.0
		DK3	25.01	28.11	4.53	35.66	1.37	94.7
		DK4	24.83	22.58	3.72	29.28	1.33	81.7
		DK5	24.18	20.93	3.65	28.85	1.32	78.9
		DK6	22.88	19.84	3.60	27.61	1.25	75.2
		S	13.64	16.72	3.39	22.30	1.26	57.3
	Palermo	WM	44.48	58.79	7.18	50.82	0.93	162.2
		DK1	36.29	51.20	6.96	40.18	0.88	135.5
		DK2	33.40	46.66	6.53	36.57	0.79	124.0
		DK3	29.18	46.51	6.43	36.24	0.70	119.1
		DK4	26.91	46.39	6.17	35.10	0.68	115.2
		DK5	24.41	41.01	6.06	25.01	0.65	97.1
		DK6	27.29	37.06	5.98	18.33	0.64	89.3
		S	7.30	28.62	4.12	16.80	0.50	57.3
Timilia	Agrigento	WM	36.04	47.21	6.68	44.74	3.38	138.1
		DK1	28.82	43.43	6.19	45.12	3.25	126.8
		DK2	25.19	41.84	5.89	35.18	3.03	111.1
		DK3	20.15	33.62	5.39	34.33	3.02	96.5
		DK4	21.25	29.99	5.13	28.75	2.70	87.8
		DK5	17.49	26.49	4.89	22.93	2.67	74.5
		DK6	18.00	25.67	4.70	22.96	2.45	73.8
		S	5.90	14.45	2.94	16.87	2.06	42.2
	Foggia	WM	57.40	49.11	5.60	50.20	1.87	164.2
		DK1	44.61	44.67	5.60	39.13	1.85	135.9
		DK2	41.85	42.43	5.22	38.76	1.75	130.0

		DK3	37.56	38.95	5.00	37.06	1.73	120.3
		DK4	33.93	34.31	5.00	36.60	1.65	111.5
		DK5	33.53	32.18	4.84	29.85	1.65	102.0
		DK6	28.25	24.95	4.51	26.91	1.51	86.1
		S	9.66	21.97	3.19	13.71	1.26	49.8
Cappelli	Palermo	WM	38.41	54.95	7.52	44.08	0.84	145.8
		DK1	31.27	45.90	6.64	33.31	0.72	111.5
		DK2	25.58	42.75	6.14	27.57	0.70	102.0
		DK3	23.37	40.36	5.89	25.69	0.69	86.1
		DK4	23.60	35.89	4.81	24.81	0.65	89.8
		DK5	22.43	28.18	4.88	24.58	0.64	80.7
		DK6	24.36	21.03	4.55	17.67	0.64	68.3
		S	11.04	17.55	3.62	14.27	0.60	47.1
	Agrigento	WM	31.74	52.30	6.17	53.80	3.92	147.9
		DK1	23.83	41.01	5.00	39.21	3.67	112.7
		DK2	19.41	35.36	4.23	29.19	3.63	91.8
		DK3	18.77	31.60	4.22	24.45	3.35	82.4
		DK4	18.46	27.39	3.73	19.35	3.11	72.0
		DK5	16.77	26.32	3.62	17.79	3.07	67.6
		DK6	14.87	23.52	3.43	14.69	2.90	59.4
		S	5.31	15.86	2.34	7.35	2.80	33.7
	Foggia	WM	49.40	44.07	7.35	58.00	1.63	160.4
		DK1	40.26	37.10	6.07	40.91	1.55	125.9
		DK2	34.72	29.40	4.47	31.75	1.48	101.8
		DK3	32.98	25.90	4.51	24.53	1.46	89.4
		DK4	28.66	22.17	3.87	20.09	1.43	76.2
		DK5	26.47	19.33	3.17	18.05	1.32	68.3
		DK6	25.71	18.56	3.36	15.06	1.31	64.0
		S	10.89	13.84	2.68	12.99	1.29	41.7

Analysis of variance	*F*	*p*	*F*	*p*	*F*	*p*	*F*	*p*	*F*	*p*	*F*	*p*
G	73.8	***	19.2	***	20.6	***	131.6	***	930.6	***	66.2	***
E	439.8	***	124.4	***	121.0	***	28.6	***	12156.4	***	57.7	***
T	356.6	***	94.5	***	88.0	***	534.3	***	93.2	***	537	***
G x E	3.0	**	5.6	***	4.5	***	30.9	***	182.3	***	9.3	***

G x T	4.9	***	1.3	ns	2.9	***	11.1	***	2.3	**	5.9	***
E x T	6.1	***	0.6	ns	0.5	ns	4.8	***	14.4	***	12	ns
G x E x T	0.8	ns	0.7	ns	0.6	ns	3.4	***	1.4	ns	1.2	ns

G, E, T and G x E, G x T, G x E x T are measured at $p < 0.01$.
Ns, not significant; *, ** and *** represents significance at $P < 0.05$, $P < 0.01$ and $P < 0.001$, respectively.
WM, wholemeal; S, semolina; DK-1 to DK-6, debranned kernels.
